# Femtosecond Laser-assisted Preparation of Conjunctival Autograft for Pterygium Surgery

**DOI:** 10.1038/s41598-020-59586-z

**Published:** 2020-02-14

**Authors:** Yu-Chi Liu, Angel Jung Se Ji, Tien-En Tan, Matthias Fuest, Jodhbir S. Mehta

**Affiliations:** 1 Tissue Engineering and Stem Cell Group, Singapore Eye Research Institute, Singapore, Singapore; 2 Department of Cornea and External Eye Disease, Singapore National Eye Centre, Singapore, Singapore; 3 Ophthalmology and Visual Sciences Academic Clinical Program, Duke-NUS Medical School, Singapore, Singapore; 4 Department of Ophthalmology, RWTH Aachen University, Aachen, Germany; 5 School of Material Science and Engineering, Nanyang Technological University, Singapore, Singapore

**Keywords:** Eye diseases, Clinical trials

## Abstract

Femtosecond laser-assisted conjunctival autografts (CAG) preparation was recently proposed. This study reports the outcomes of the first clinical trial on the use of laser to prepare CAG in pterygium surgery, and to compare the outcomes with those of manual technique. Forty eyes undergoing primary pterygium excision with laser-assisted CAG transplantation were prospectively included (L group). Two historical matched cohorts whose CAGs were prepared manually were compared (n = 78 eyes by the same experienced surgeon, M group; n = 78 eyes by trainees; TM group). We found the laser-created CAGs had only 11 μm deviation from the targeted thickness. The best-corrected visual acuity improved, and the astigmatism significantly decreased after surgery, with comparable efficacy across 3 groups. The 1-year recurrence rate was 2.5%, 3.8% and 7.7% in the L, M and TM groups, respectively (*P* = 0.12). There was no significant difference between the L and M groups in the complication rate (5.0% and 1.3%, respectively), surgical time (19.4 ± 5.1 and 19.1 ± 6.2 minutes, respectively), and postoperative discomfort scores (0.1 ± 0.3 and 0.2 ± 0.3, respectively), but these outcomes were significantly less favorable in the TM group. The results of this first comparative clinical trial suggest that femtosecond laser-assisted CAG preparation can be considered as an alternative technique for CAGs preparation.

## Introduction

Pterygia is a common ocular surface disease characterized by fibrovascular growth arising from the conjunctiva and extending onto the cornea. The prevalence rates of pterygia range from 3% to 19.5%^1^, with a higher rate in men and in countries closer to the equator^1,2^. Surgical removal is the main treatment for pterygia, and the surgical techniques can be divided into 3 types: (1) Bare sclera technique, in which no tissue grafting is used after pterygium excision (2) Simple closure, and (3) Tissue grafting technique, in which a conjunctival autograft (CAG), limbal conjunctival autograft, or amniotic membrane transplantation (AMT) is used to cover the area from which the pterygium is excised^3,4^. The surgery is minimally invasive, however, the recurrence rate can be as high as 88% in certain populations and is surgical technique dependent^5^.

The risk of recurrence after pterygium surgery is multifactorial, with patient-attributed factors, such as age, ethnicity, morphology of pterygium^3,6,7^, and surgeon-attributed factors, such as the surgical experiences and techniques^3,8^. A meta-analysis of randomized controlled trials has shown that bare scleral technique increases the risk of postoperative recurrence by 6 to 25 times compared to a tissue grafting technique^9^. Among the tissue grafting options, CAG is associated with a lower risk of recurrence than AMT, and patients with recurrent pterygium are at particular lower risk of recurrence when they receive CAG than AMT^10^. CAG is also associated with minor complications and excellent cosmetic outcomes^3,11,12^. In addition to surgical techniques, the activity of conjunctival stromal fibroblasts during the wound healing after pterygium surgery, is thought to be a main contributing factor in the development of recurrence^13^, hence minimal inclusion of Tenon’s tissue, which is composed of fibroblasts^14^, during the CAG harvesting, can minimize recurrence^15^. However, dissecting a tenon-free CAG is highly technically dependent and can be time-consuming, especially for novice surgeons^11,15^.

Femtosecond lasers have been applied to the field of refractive surgery^16^, cataract surgery^17^ and corneal transplantation^18,19^, providing reproducible, accurate and reliable capsulotomy^20^, corneal flaps, stromal lenticules^21^, or corneal grafts^18,19^. In order to attempt to transfer these favorable characteristics of femtosecond lasers to pterygium surgery, recently, we described a new technique, in which a low-energy, high-frequency femtosecond laser (Z8 LDV, Ziemer Ophthalmic Systems AG, Port, Switzerland) was used to prepare CAGs in pterygium surgery^22,23^. The feasibility, reproducibility and optimization of laser settings were firstly tested using porcine eyes, and the results showed that the femtosecond laser allowed accurate and reliable preparation of ultra-thin CAGs, with consistent achieved thickness of 60 μm. The deviation from the targeted CAG size was also low at 7.6%, and the technique was independent of surgeons’ experience^23^. In the subsequent clinical pilot study on 5 patients, we reported that the use of a femtosecond laser was a fast and reliable method to prepare ultrathin CAGs. The achieved CAGs were consistently uniform and thin, and no intraoperative complications such as buttonholes or CAGs occurred. During the one-month follow-up, no postoperative complications or recurrences were observed^22^. Moreover, there was no collateral thermal damage resulting from the laser pulses seen in the histological sections on the CAGs^24^, and the vessel revascularization over the whole CAG was complete within 3 months after transplantation^25^. With the increasing popularity of femtosecond lasers in modern anterior segment surgery, femtosecond laser-assisted CAGs preparation can be an additional application in centers that are already equipped with a laser. However, studies with a larger cohort and longer follow-up are required. In this study, we report the 1-year surgical outcomes of pterygium excision with femtosecond laser-assisted CAG. This is the first clinical study presenting the outcomes regarding this new technique. We also compared the clinical outcomes with those of manually-prepared CAGs.

## Results

### Patients characteristics

The mean age at the time of surgery was 62.6 ± 11.8, 62.2 ± 13.4 and 64.7 ± 10.3 years, for the L, M and TM groups, respectively (*P* = 0.13). The patients’ age, gender, race and laterality of surgery were comparable across the groups. The patients’ population was mainly Chinese and was male dominant. As grade T3 pterygia have been shown to be associated with postoperative recurrence^26^, the pterygia were categorized into T3 or non-T3, with a comparable proportion of T3 pterygia among the three groups (32.5%, 33.3% and 37.2% for the L, M and TM groups, respectively; *P* = 0.57). The patients’ characteristics are summarized in Table 1.

**Table 1:** Patients and pterygia characteristics.

	Femtosecond laser group (n = 40 eyes)	Manual group (n = 78 eyes)	Trainees manual group (n = 78 eyes)	*P* value
Mean age (years)	62.6 ± 11.8	62.2 ± 13.4	64.7 ± 10.3	0.13
Gender (male)	72.5%	69.1%	81.6%	0.33
Laterality (right)	50.0%	52.5%	47.1%	0.85
Race (Chinese^†^)	80.0%	74.5%	83.7%	0.51
Pterygium grade (T3) (T3:T2:T1)	32.5% (13:21:6)	33.3% (26:41:11)	37.2% (29:39:10)	0.57

^†^Other ethnical groups include Malays, Indians, Caucasians, Filipinos and Burmese.

### Intraoperative and postoperative outcomes

For the L, M and TM groups, the preoperative logMAR BCVA was 0.21 ± 0.17, 0.26 ± 0.22 and 0.30 ± 0.19, respectively (*P* = 0.10). The postoperative BCVA at 3 months was 0.18 ± 0.18, 0.23 ± 0.20 and 0.28 ± 0.25, respectively (*P* = 0.15). The changes between the preoperative and postoperative 3-month logMAR BCVA was not significant (*P* = 0.38, *P* = 0.95 and *P* = 0.61, respectively). The preoperative astigmatism was 2.38 ± 1.44, 2.07 ± 1.91 and 2.89 ± 2.27 diopters (D), respectively (*P* = 0.25), and the postoperative 3-month astigmatism was 1.74 ± 0.89, 1.05 ± 0.96 and 1.77 ± 1.29 D, respectively (*P* = 0.25). The changes between preoperative and postoperative 3-month astigmatism was statistically significant in all groups (*P* = 0.003, *P* = 0.002 and *P* < 0.001, respectively). Table 2 presents the results of vector analyses. For the L, M and TM groups, the preoperative J_0_ was 0.35 ± 0.48, 0.33 ± 0.51 and 0.40 ± 0.48, respectively (*P* = 0.61), indicating that the pterygia resulted in with-the-rule astigmatism. After surgery, the extent of with-the-rule astigmatism decreased, even slightly towards against-the-rule astigmatism in the L group. At 3 months, the J_0_ value was −0.01 ± 0.23, 0.00 ± 0.39 and 0.08 ± 0.28, respectively (*P* = 0.37). The oblique astigmatism also reduced after surgery in all groups (Table 2). The efficacy on reducing the cylindrical power and astigmatic vectors was comparable across the groups (*P* = 0.41, *P* = 0.25 and *P* = 0.28, for the changes in astigmatism, J_0_ and J_45_, respectively).

**Table 2:** Preoperative and postoperative vector values of refractive astigmatism for 3 groups.

	Femtosecond laser group	Manual group	Trainees manual group	*P* value
Preoperative J_0_	0.35 ± 0.48	0.33 ± 0.51	0.40 ± 0.48	0.61
Preoperative J_45_	0.76 ± 0.72	0.68 ± 0.71	0.84 ± 0.82	0.29
Postoperative 3-months J_0_	−0.01 ± 0.23	0.00 ± 0.39	0.08 ± 0.28	0.37
Postoperative 3-months J_45_	−0.14 ± 0.18	−0.26 ± 0.19	0.11 ± 0.19	0.30

**Figure 1:**
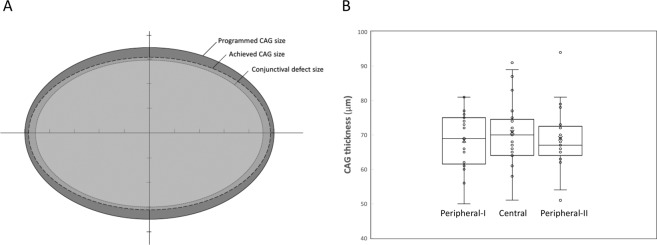
Diagram illustration showing the conjunctival defect area after pterygium excision (dotted line), programmed CAG size (solid line), and achieved CAG size measured immediately after laser cutting (long-dashed line). (**A**) The achieved CAG size was slightly smaller than the programmed CAG size because of the tissue contraction resulting from the elasticity of conjunctiva, but it still larger than the conjunctival defect area. Box plot showing the central thickness and peripheral thickness 2 mm from the center on each side, of the CAGs. (**B**) The CAGs achieved were planar. The central rectangle represents the first quartile to the third quartile, a segment inside shows the median, “whiskers” above and below the box show the minimum and maximum, and cross signs indicate the mean values.

**Figure 2:**
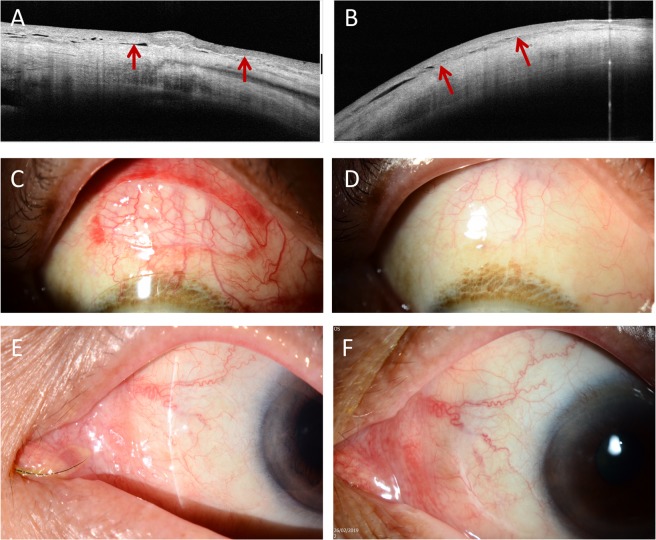
Representative ASOCT and slit lamp pictures showing the femtosecond laser-assisted CAG preparation. ASOCT pictures showing the transplanted CAG at 1 (**A**) and 3 months (**B**) postoperatively (arrows). The graft edema resolved with time. Slit lamp pictures showing the CAG harvested area at 1 day (**C**) and 1 week (**D**), and the area of CAG transplantation at 3 months (**E**) and 12 months (**F**) postoperatively. The conjunctival epithelium at the harvested site healed within a mean time of 1 week with no evidence of scarring. There was no occurrence of graft ischemia, graft dehiscence or granuloma formation at the CAG transplantation area.

When examining the femtosecond laser-assisted technique specifically, the laser resection of the CAG took 21 seconds to complete. The mean conjunctival defect size was 40.8 ± 7.6 mm^2^, and the mean CAG area measured after laser cutting was 46.1 ± 5.9 mm^2^ (Fig. 1A). The mean graft thickness measured immediately after laser cutting was 70.9 ± 10.3 μm at the center, and were 68.4 ± 7.9 μm and 69.0 ± 8.7 μm 2 mm from the center on each side (Fig. 1B). The time to remove the CAG from the conjunctival stromal bed was 4.7 ± 2.8 seconds. Graft edema resolved with time (Fig. 2A,B), with the thickness decreasing from 199.9 ± 73.9 μm at 1 week, to 144.1 ± 29.4 μm at 12 months postoperatively (Table 3). The conjunctival epithelium at the harvested site healed within a mean time of 1 week with no evidence of conjunctival scarring in all cases (Fig. 2C,D). The conjunctival thickness at the harvested area also decreased with time, from 298.2 ± 122.4 μm at 1 week, to 248.0 ± 44.5 μm at 12 months (Table 3).

**Table 3:** Changes in the conjunctival thickness at the harvested site and CAG thickness in the femtosecond laser-assisted CAG preparation.

Postoperative timepoint	CAG thickness (μm)	Conjunctival thickness (μm)
1 week	199.9 ± 73.9	298.2 ± 122.4
1 month	182.7 ± 89.0	262.7 ± 67.8
3 months	159.3 ± 80.2	246.7 ± 56.7
6 months	162.1 ± 66.8	248.6 ± 77.9
12 months	144.1 ± 29.4	248.0 ± 44.5

There was a significant difference in the surgical time between the TM group (23.5 ± 5.9 minutes) and L group (19.4 ± 5.1 minutes, *P* = 0.008), as well as the manual group (19.1 ± 6.2 minutes, *P* = 0.001). Manual CAG preparations performed by trainees were also associated with significantly higher postoperative discomfort scores (1.1 ± 0.7), compared to that in the L (0.1 ± 0.3; *P* = 0.02) and M groups (0.2 ± 0.3; *P* = 0.03) performed by the experienced surgeon. When comparing laser-assisted versus manually-prepared technique by the same surgeon, there was no significant difference in the surgical time and postoperative discomfort scores (*P* = 0.73 and *P* = 0.86, respectively).

In the L group, there was 1 case with a buttonhole on the CAG and a case with an incomplete laser side cut, which was completed manually subsequently (complication rate: 2/40 = 5.0%). There were no other intraoperative or postoperative complications (Fig. 2E,F). There was 1 graft dehiscence in the M group (1/78 = 1.3%). When trainees prepared the CAGs manually, 4 cases had CAG buttonholes and 3 cases had postoperative graft dehiscence (complication rate: 7/78 = 9.0%). The complication rate in the TM group was significantly higher than that in the L and M group (*P* = 0.03 and *P* = 0.01, respectively). The 1-year recurrence rate was 2.5%, 3.8% and 7.7% for the L, M and TM groups. Surgeries performed by trainees had a borderline significantly higher recurrence rate than the other two groups (*P* = 0.06). There was no significant difference between the L and M groups (*P* = 0.88).

## Discussion

In the present study, we reported the surgical outcomes of femtosecond laser-assisted CAG transplantation in pterygium surgery. It allowed accurate and reliable preparation of tenon-free CAGs which were consistently ultra-thin. The visual and refractive outcomes were comparable to those of manual technique, and it was associated with a low recurrence rate. The laser-assisted technique did not prolong the surgical time. Patients’ postoperative discomfort and the occurrence of intra- or postoperative complications were more related to surgeons’ experiences, rather than the technique used (manual or laser). Although laser-assisted CAG preparation may be limited by the cost and availability of laser machine, the aim of this study was to report the clinical outcomes of the first clinical trial on this new technique. For institutes that are already equipped with a laser, laser-assisted conjunctival autografting can be an additional application.

Biological tissue grafts have become an important part of the surgical management of pterygia. Among the tissue grafting options, CAGs have emerged as the technique of choice, compared to bare sclera and AMT due to lower recurrence rates and better cosmetic appearances^3^. When comparing to limbal conjunctival autografts, there was no difference with respect to recurrence, for primary pterygia^27^. However, manual CAG preparation, represents a difficult and time-consuming part of the conventional pterygium surgical procedure, particularly the dissection of the conjunctiva with minimal inclusion of Tenon’s tissue. We have demonstrated in this study that the femtosecond laser allowed precise creation of CAGs. The CAG thickness was uniform, and the deviation was only 11 μm from the targeted thickness. As the average bulbar conjunctival epithelial thickness is approximately 54.7 μm^28^, the CAGs prepared by the laser in the present study included only minimal tenon’s tissue, which is favorable for reducing postoperative recurrence and graft retraction^15^. Studies have shown that achieving an ultra-thin CAG requires considerable surgical skill and is associated with a substantial learning curve^11,15^: approximately 50 attempts were required for a trainee to achieve a CAG thickness of 87 μm^11^. We have previously shown that the laser can accurately cut a CAGs, independent of surgeon experience^23^, hence, the laser technique may be valuable in overcoming the learning curve for preparation of thin or ultra-thin CAGs.

Among the available femtosecond laser platforms, the large numerical aperture of the Ziemer Z8 system enables it to cut through translucent tissue like conjunctiva^22,23^. We have previously reported a case in which the Z8 laser was used to excise a conjunctival melanosis^24^. As conjunctival tissue is less transparent than corneal tissue and the energy penetrance therefore would be lower in conjunctiva, the energy setting for the conjunctival stromal lamellar cut was slightly higher than that for normal corneal stromal lamellar cut (100% versus 90%). This increase in the energy did not result in thermal damage in histological sections of the conjunctiva^24^. Moreover, the Ziemer femtosecond laser, compared to higher-energy platforms, delivers spot energy in 1–5 nanojoules range (as opposed to microjoules), resulting in less tissue inflammation^29^, which is an important favorable aspect in conjunctival surgery as it can minimize conjunctival scarring. In our cases, we did not observe any conjunctival scarring or delayed healing of the conjunctival epithelium at the harvested area. The conjunctival edema resolved with time and returned to a normal range after 1 month postoperatively (Table 3)^30^. We have also previously evaluated the conjunctival re-reperfusion rate at the harvested site using OCT angiography (OCTA). The conjunctival vascularization was restored at 1 month, and the underlying episcleral vascular network was not affected by the laser^25^. In addition, the Z8 laser also enabled easy applanation to the superior bulbar surface for CAG harvesting because the handpiece can be freely positioned to various angles with the easily maneuverable articulating hand piece, and the dissection required no suction, avoiding intraoperative complications such as elevated intraocular pressure.

There is currently no specific laser module for CAG harvesting, and the mode we used was the lamellar keratoplasty module. The laser dissection can be customized to a wide range of programmed diameters (5–10 mm) and depths (60 to 2000 μm). After the laser cutting, there was some shrinkage of the CAG because of the tissue contraction resulting from the elasticity of conjunctiva (targeted: 55.0 mm^2^; achieved 46.1 mm^2^), but the post-cut CAG area was still larger than the conjunctival defect area (46.1 versus 40.8 mm^2^). The transplanted CAGs had some graft swelling postoperatively but resolved with time. Compared to a previously published study, in which the thickness of manually-prepared CAGs was reported to be 291 ± 124 μm at 3 months postoperatively^30^. Our study showed thinner CAG thickness (159.3 ± 80.2 μm) with the laser technique. This difference might be due to inclusion of Tenon’s tissue in the manual dissection. The CAG thickness continued to decrease with time, with the thickness at 144.1 ± 29.4 μm at 12 months. The difference between the measured thickness and targeted thickness (60 μm) might result from these reasons: (1) The margin of the CAG became indiscernible with time because of the resolution of tissue edema and wound healing, hence there might be an overestimation in the measurements (2) Residual graft edema (3) Residual fibrin glue at the interface (4) Proliferation of residual tenon on the CAG (5) Limitation of the image resolution of the RTVue system (5 μm at superior and inferior graft margin each)^31,32^. A potential concern on using ultra-thin CAGs is graft re-vascularization, as the blood flow and oxygenation may be better preserved to a greater extent in thicker grafts^33^. However, our previous OCTA study has demonstrated that the vessel regrowth into the CAGs started as early as 1 week postoperatively, the CAGs became well-perfused at 1 month, and re-vascularization was completed at 3 months^25^. There was no postoperative graft ischemia or necrosis seen in the L group in the present study.

With regard to the visual and refractive outcomes, there was an improvement in the BCVA after surgery in all groups, although it was not statistically significant. Pterygia induced astigmatism (mainly with-the-rule astigmatism in the present study population) and noticeably high J_0_ and J_45_ values, compared to the normal elderly population who have no pterygia^34^. After surgery, the cylindrical power, as well as the extent of with-the-rule or oblique astigmatism significantly decreased, and the amplitude of this decrease was not significantly different between the L and M groups. These findings are similar to previous studies in the literature, where authors have shown that the types of grafts (CAG, AMT or conjunctival rotational flap) did not have a significant effect on the change in the degree of astigmatism^35^. In addition, it has been shown that the refractive changes, including the spherical power, astigmatism and surface regularity, stabilized 1 month after pterygium surgery^36^. Hence, we included the visual and refractive outcomes up to postoperative 3 months only for the analysis, as some of the patients underwent subsequent cataract extraction that might affect the analysis.

In the L group, there were one case with an incomplete side cut and one case with a buttonhole on the graft. This might result from the learning curve effects. As the laser docking was suction-free, it is crucial that surgeons need to hold the handpiece steady to ensure even applanation of glass contact lens on the conjunctival surface and resultant even laser photodisruption, avoiding the occurrence of uncut or an incomplete cut.

Hirst *et al*. reported that after surgery, 50% of recurrence occurred within 4 months, and 97% occurred within 12 months, suggesting one-year follow-up in the present study is an adequate time to assess the recurrence^37^. We found that the 1-year recurrence rate was low with CAG transplantation, regardless of the method used for CAG harvesting (laser: 2.5%, manual: 3.8%), when surgery was performed by an experienced surgeon. The L group had a lower recurrence rate than the M group, but it was not statistically significant. Surgery performed by trainees was associated with borderline significantly higher postoperative recurrence (7.7%), which was consistent with previous studies^8,15^. Previous reported recurrence rate was 3.3–18.5% for experienced surgeons^6^, and 10–82% for trainees^8,15^. The lower recurrence rate in this study can be attributed to two reasons: CAG transplantation has been shown to be superior to bare sclera technique or AMT^4,9,10^, with respect to recurrence, and in all cases we used fibrin glue, which is associated with lower recurrence compared to sutures^38^.

There are some limitations in this study. Firstly, data for the two manual groups were collected as a historical cohort, while the laser group was recruited prospectively. The former might result in an underestimation of intraoperative or postoperative complications if they were not well documented in the medical records. Some variables such as CAG preparation time and keratometric data were not available in the historical manual groups as the recruitment was not in a prospective manner. Secondly, as the laser-assisted CAG creation is a relatively new technique, this treatment option was only available for consultant-level surgeons in our center. Hence the group of “laser technique performed by trainees” is lacking, and this will be included in future studies to evaluate the added value of the laser technique to shorten the learning curve of CAGs preparation. Comparing the CAG preparation time specifically, rather than the surgical time, in future studies, will also help better understand whether the laser technique has beneficial effects with regard to CAG preparation time.

In conclusion, we have demonstrated that the femtosecond laser allows reliable, accurate and consistent ultra-thin CAGs preparation. It was associated with low complication rate, low recurrence rate, minimal discomfort, good cosmetic appearance, and comparable surgical time as well as visual and refractive outcomes to conventional manual technique. As the overall utilization of lasers in ophthalmic surgery is growing, for institutes that are already equipped with a laser, laser-assisted CAG preparation, in pterygium surgery or other conjunctival surgery, can be an extended application.

## Methods

### Patients, surgical technique and intraoperative parameters

Forty eyes of thirty-eight consecutive patients with primary pterygium were prospectively included and received pterygium excision with femtosecond laser-assisted CAG transplantation (laser (L) group). The preoperative pterygium was graded based on the relative translucency of pterygium tissue^26^: T1: a pterygium in which episcleral vessels underlying the pterygium body were clearly distinguished; T3: a pterygium in which episcleral vessels underlying the pterygium body were totally obscured; T2: all other pterygia that did not fall into T1 or T3.

**Figure 3:**
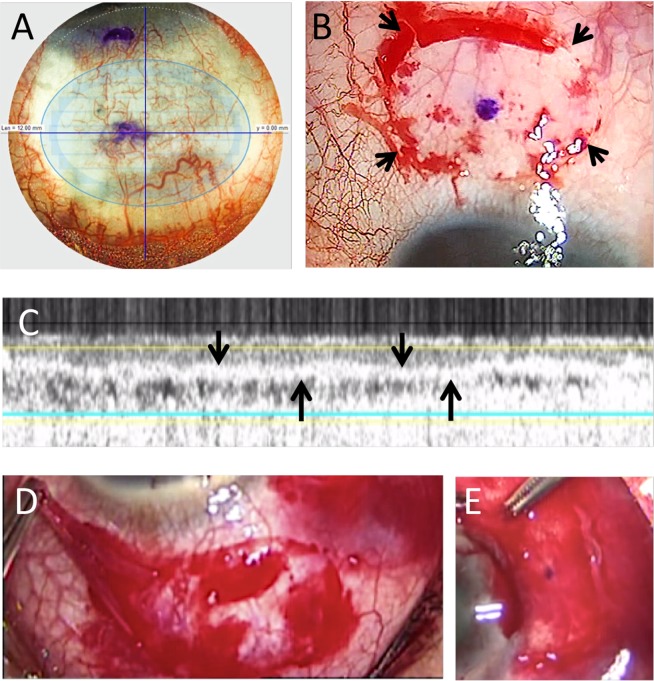
Pterygium excision with femtosecond laser-assisted conjunctival autograft (CAG) transplantation. The laser was programmed to harvest an ellipsoid CAG of 7 × 10-mm diameter and 60-μm depth from superior bulbar conjunctiva. (**A**) Outline of the CAG (arrows) right after the laser cutting. (**B**) In-built intraoperative OCT showing complete lamellar dissection of CAG (arrows). The CAG thickness was also measured immediately after laser cutting. (**C**) The CAG was lifted from the conjunctival stromal bed. (**D**) The CAG was positioned and glued onto the area of conjunctival defect (**E**).

The surgery was performed with a technique as we described previously^22,23^. Under local anesthesia, a 6-0 vicryl traction suture (Ethicon, Somerville, MA) was firstly placed in the peripheral superior cornea to control the eye position. The pterygium was separated from the underlying sclera by blunt dissection. The body of the pterygium was excised at the level of the limbus, and the head of the pterygium was removed from the corneal surface with a Mini-Blade. The remaining pterygium and subconjunctival Tenon’s tissue were removed to expose the bare sclera, and the resultant area of conjunctival defect was measured by a caliper. The eyeball was then rotated down using the traction suture to maximize the exposed area of superior bulbar conjunctiva. The center of the planned CAG harvesting area was marked, which was approximately 4 mm away from the limbus, so that the edge of the CAGs was close to the limbus. The laser handpiece was then placed onto the bulbar conjunctival surface. As no suction was used, it was important for the surgeon to hold the handpiece steady, with good docking centration by placing the conjunctival marking in the middle of the crosshair on the laser visualization screen (Fig. 3A). The laser head was then gently applanated on the bulbar conjunctiva, and a symmetrical appearance of the tear meniscus going beyond the programmed resection area, shown on the screen display, indicated good applanation. Subsequently, the Ziemer Z8 laser resection was activated. In all cases, the laser was programmed to harvest an ellipsoid CAG of 7 × 10-mm diameter (55.0 mm^2^) and 60-μm depth using the lamellar keratoplasty module (Fig. 3B). Laser energy was set at 100% for the conjunctival stromal lamellar cut and 130% for the side cuts. Immediately after the laser resection, the CAG thickness was measured by the in-built optical coherence tomography (OCT) at the CAG center and 2 mm from the center on either side (Fig. 3C). The laser head was then lifted off the conjunctival surface, and the dimensions of the CAG was measured with a caliper. The CAG was then gently peeled from the conjunctival stromal bed using forceps (Fig. 3D), and the time of this removal was recorded. The CAG was then positioned and glued onto the area of the conjunctival defect with fibrin glue (Fig. 3E; Artiss; Baxter, Westlake Village, CA). A bandage contact lens was applied at the end of surgery. All the surgery was performed by a single experienced surgeon (JSM). This study was a registered controlled trial (NCT02866968). Approval for the study was granted by the institutional review board of SingHealth, Singapore (number: 2016/2512), and the study was conducted in accordance to the Declaration of Helsinki. The informed consent was obtained from each patient.

An independent historical cohort of 78 patients with primary pterygium who underwent pterygium excision with manually-prepared CAG transplantation performed by the same surgeon, were compared (manual (M) group). These patients were age- and pterygium grade-matched to the L group. The surgical procedure for the pterygium excision was identical to that in the L group, but the CAG harvesting was created manually, using vannas scissors, with as minimal inclusion of tenon’s tissue as possible. The CAG was then glued onto the area of conjunctival defect with fibrin glue. For both L and M groups, the postoperative regimen consisted of topical preservative-free dexamethasone and levofloxacin every 3 hours for 1 week, on a tapering dose over 8 weeks.

To evaluate the effects of surgical experiences on the harvesting of CAGs, another historical independent cohort of 78 patients with primary pterygium who underwent pterygium excision with manually-prepared CAG transplantation with fibrin glue, performed by trainees during the period of 2015–2017, were also included (trainees manual (TM) group). These patients were age- and pterygium-grade matched to the L and M groups.

### Clinical evaluation and data collection

The following data were collected and analyzed: age, gender, race, best-corrected visual acuity (BCVA) and cylindrical power of automatic refraction before surgery and at 3 months postoperatively, surgical time, patients discomfort on the first day after surgery (from 1: least discomfort, to 10: most discomfort), intraoperative and postoperative complications, and 1-year recurrence rate, defined as fibrovascular regrowth onto the limbus. The BCVA was converted to logarithm of the minimum angle of resolution (logMAR) values, and a vector analysis was used to analyze the changes of cylindrical power before and after surgery^34^: J_0_ = −C/2 × cos 2α; J_45_ = −C/2 × sin 2α, where C was negative cylindrical power and α was cylindrical axis. J_0_ refers to with-the-rule (positive values) or against-the-rule astigmatism (negative values), and J_45_ refers to oblique astigmatism. None of the patients received cataract extraction surgery that might affect the visual and refractive analyses within 3 months after the pterygium surgery. For the L group, the CAG thickness 1 and 3 mm away from the limbus, and the conjunctival thickness at the center and 2 mm on each side at the harvested area, were measured by an independent observer (TET) with the in-built caliper of anterior segment OCT (ASOCT; RTVue; Optovue, Inc., Fremont, CA, USA), at 1 week, 1, 3, 6 and 12 months postoperatively. Three measurements were taken for each eye at each time point, and the average was used for analysis.

### Statistical analysis

The required sample size was calculated by assuming that the 1-year recurrence rate was 10% in the M group based on a review of current literature^3–6,8–10,12,26^, and the odds ratio of recurrence between the M and L groups was 1.5 based on our preliminary data on the laser-assisted CAGs preparation. A sampling ratio of 1:2:2 for the L, M and TM groups was used to add statistical power^39^. Thus, a sample of 37 subjects per group was sufficient to detect the difference with a power of ≥80% and at a 5% significance. Known confounders including age and pterygium grades were matched for the M and TM groups.

All data were expressed as mean ± standard deviation. The comparisons among the three groups were performed using a one-way analysis of variance with a Bonferroni correction. Chi-square analyses were used to calculate the proportional difference among the groups. A paired t-test was used to compare the preoperative and postoperative values. The statistical analysis was carried out using STATA; STATACrop, College Station, TX), and a *P* < 0.05 was considered as significant.

## References

[1] Rezvan F (2018). Prevalence and risk factors of pterygium: a systematic review and meta-analysis. Surv. Ophthalmol..

[2] Hovanesian JA (2017). Surgical techniques and adjuvants for the management of primary and recurrent pterygia. J. Cataract. Refract. Surg..

[3] Janson BJ, Sikder S (2014). Surgical management of pterygium. Ocul. Surf..

[4] Clearfield E, Hawkins BS, Kuo IC (2017). Conjunctival autograft versus amniotic membrane transplantation for treatment of pterygium: Findings from a cochrane systematic review. Am. J. Ophthalmol..

[5] Chen PP, Ariyasu RG, Kaza V, LaBree LD, McDonnell PJ (1995). A randomized trial comparing mitomycin C and conjunctival autograft after excision of primary pterygium. Am. J. Ophthalmol..

[6] Nuzzi R, Tridico F (2018). How to minimize pterygium recurrence rates: clinical perspectives. Clin. Ophthalmol..

[7] Hirst LW, Smallcombe K (2017). Double-Headed Pterygia Treated With P.E.R.F.E.C.T for PTERYGIUM. Cornea.

[8] Farrah JJ, Lee GA, Greenrod E, Vieira J (2006). Outcomes of autoconjunctival grafting for primary pterygia when performed by consultant compared with trainee ophthalmologists. Clin. Exp. Ophthalmol..

[9] Sanchez-Thorin JC, Rocha G, Yelin JB (1998). Meta-analysis on the recurrence rates after bare sclera resection with and without mitomycin C use and conjunctival autograft placement in surgery for primary pterygium. Br. J. Ophthalmol..

[10] Clearfield E, Muthappan V, Wang X, Kuo IC (2016). Conjunctival autograft for pterygium. Cochrane Database Syst. Rev..

[11] Kuo MX, Sarris M, Coroneo MT (2015). Cadaveric porcine model for teaching and practicing conjunctival autograft creation. Cornea.

[12] Ang LP, Chua JL, Tan DT (2007). Current concepts and techniques in pterygium treatment. Curr. Opin. Ophthalmol..

[13] Kim KW, Park SH, Kim JC (2016). Fibroblast biology in pterygia. Exp. Eye Res..

[14] Kakizaki H (2012). Anatomy of Tenons capsule. Clin. Exp. Ophthalmol..

[15] Ti SE, Chee SP, Dear KB, Tan DT (2000). Analysis of variation in success rates in conjunctival autografting for primary and recurrent pterygium. Br. J. Ophthalmol..

[16] Yc, L., Ak, R. & J, M. In *Cornea* Vol. 2 (eds Krachmer, J. H., Mannis, M. J. & Holland, E. J.) (Elsevier Mosby, 2016).

[17] Liu YC, Setiawan M, Ang M, Yam GHF, Mehta JS (2019). Changes in aqueous oxidative stress, prostaglandins, and cytokines: Comparisons of low-energy femtosecond laser-assisted cataract surgery versus conventional phacoemulsification. J. Cataract. Refract. Surg..

[18] Liu YC (2014). Endothelial approach ultrathin corneal grafts prepared by femtosecond laser for descemet stripping endothelial keratoplasty. Invest. Ophthalmol. Vis. Sci..

[19] Liu YC (2019). Intraoperative optical coherence tomography-guided femtosecond laser-assisted deep anterior lamellar keratoplasty. Cornea.

[20] Popovic M, Campos-Moller X, Schlenker MB, Ahmed II (2016). Efficacy and safety of femtosecond laser-assisted cataract surgery compared with manual cataract surgery: A meta-analysis of 14 567 Eyes. Ophthalmology..

[21] Hall RC, Mohamed FK, Htoon HM, Tan DT, Mehta JS (2011). Laser *in situ* keratomileusis flap measurements: Comparison between observers and between spectral-domain and time-domain anterior segment optical coherence tomography. J. Cataract. Refract. Surg..

[22] Fuest M, Liu YC, Coroneo MT, Mehta JS (2017). Femtosecond laser assisted pterygium surgery. Cornea..

[23] Fuest M (2017). Femtosecond laser-assisted conjunctival autograft preparation for pterygium surgery. Ocul. Surf..

[24] Tey ML, Liu YC, Chan AS, Mehta JS (2018). Excision of conjunctival melanosis and conjunctival autografting by femtosecond laser. Clin. Exp. Ophthalmol..

[25] Liu Yu-Chi, Devarajan Kavya, Tan Tien-En, Ang Marcus, Mehta Jodhbir S. (2019). Optical Coherence Tomography Angiography for Evaluation of Reperfusion After Pterygium Surgery. American Journal of Ophthalmology.

[26] Tan DT, Chee SP, Dear KB, Lim AS (1997). Effect of pterygium morphology on pterygium recurrence in a controlled trial comparing conjunctival autografting with bare sclera excision. Arch. Ophthalmol..

[27] Al Fayez MF (2002). Limbal versus conjunctival autograft transplantation for advanced and recurrent pterygium. Ophthalmology..

[28] Feng Y, Simpson TL (2008). Corneal, limbal, and conjunctival epithelial thickness from optical coherence tomography. Optom. Vis. Sci..

[29] Riau AK (2014). Comparative study of nJ- and muJ-energy level femtosecond lasers: evaluation of flap adhesion strength, stromal bed quality, and tissue responses. Invest. Ophthalmol. Vis. Sci..

[30] Zhang X (2013). Bulbar conjunctival thickness measurements with optical coherence tomography in healthy chinese subjects. Invest. Ophthalmol. Vis. Sci..

[31] Liu YC, Lwin NC, Chan NS, Mehta JS (2014). Use of anterior segment optical coherence tomography to predict corneal graft rejection in small animal models. Invest. Ophthalmol. Vis. Sci..

[32] Sharma R (2014). Application of anterior segment optical coherence tomography in glaucoma. Surv. Ophthalmol..

[33] Memarzadeh K, Sheikh R, Blohme J, Torbrand C, Malmsjo M (2016). Perfusion and oxygenation of random advancement skin flaps depend more on the length and thickness of the flap than on the width to length ratio. Eplasty..

[34] Liu YC (2011). Power vector analysis of refractive, corneal, and internal astigmatism in an elderly Chinese population: the Shihpai Eye Study. Invest. Ophthalmol. Vis. Sci..

[35] Altan-Yaycioglu R, Kucukerdonmez C, Karalezli A, Corak F, Akova YA (2013). Astigmatic changes following pterygium removal: comparison of 5 different methods. Indian. J. Ophthalmol..

[36] Tomidokoro A (2000). Effects of pterygium on corneal spherical power and astigmatism. Ophthalmology..

[37] Hirst LW, Sebban A, Chant D (1994). Pterygium recurrence time. Ophthalmology..

[38] Zloto O, Greenbaum E, Fabian ID, Ben Simon GJ (2017). Evicel versus tisseel versus sutures for attaching conjunctival autograft in pterygium surgery: A prospective comparative clinical study. Ophthalmology..

[39] Lewallen S, Courtright P (1998). Epidemiology in practice: case-control studies. Community Eye Health..

